# Deficiency of macrophage migration inhibitory factor attenuates tau hyperphosphorylation in mouse models of Alzheimer’s disease

**DOI:** 10.1186/s12974-015-0396-3

**Published:** 2015-09-17

**Authors:** Shu-Qin Li, Yang Yu, Jin-Zhao Han, Ding Wang, Jin Liu, Feng Qian, Guo-Huang Fan, Richard Bucala, Richard D. Ye

**Affiliations:** School of Pharmacy, Shanghai Jiao Tong University, Shanghai, 200240 China; GlaxoSmithKline Research and Development Center, Shanghai, 201203 China; Department of Medicine, Yale University School of Medicine, New Haven, CT 06520 USA; Institute of Chinese Medical Sciences, University of Macau, Macau, SAR China

**Keywords:** Alzheimer’s disease, Macrophage migration inhibitory factor, Tau phosphorylation, Astrocyte activation

## Abstract

**Background:**

Pathological features of Alzheimer’s disease (AD) include aggregation of amyloid beta (Aβ) and tau protein. Macrophage migration inhibitory factor (MIF), a proinflammatory cytokine, has been implicated in the toxicity of aggregated Aβ. It remains unclear whether MIF affects hyperphosphorylation and aggregation of tau.

**Methods:**

The effects of MIF deficiency in tau hyperphosphorylation were examined in *Mif*^*−/−*^ mice receiving intracerebroventricular (ICV) injection of streptozotocin (STZ) and in APP/PS1 transgenic mice mated with *Mif*^*−/−*^ mice. MIF expression and astrocyte activation were evaluated in ICV-STZ mice using immunofluorescence staining. Cultured primary astrocytes were treated with high glucose to mimic STZ function in vitro, and the condition medium (CM) was collected. The level of tau hyperphosphorylation in neurons treated with the astrocyte CM was determined using Western blotting.

**Results:**

MIF deficiency attenuated tau hyperphosphorylation in mice. ICV injection of STZ increased astrocyte activation and MIF expression in the hippocampus. MIF deficiency attenuated astrocyte activation in ICV-STZ mice. CM from high glucose-treated WT astrocytes increased tau hyperphosphorylation in cultured primary neurons, an effect absent from *Mif*^*−/−*^ astrocytes and WT astrocytes treated with the MIF inhibitor ISO-1. ISO-1 had no direct effect on tau phosphorylation in cultured primary neurons.

**Conclusions:**

These results suggest that MIF deficiency is associated with reduced astrocyte activation and tau hyperphosphorylation in the mouse AD models tested. Inhibition of MIF and MIF-induced astrocyte activation may be useful in AD prevention and therapy.

**Electronic supplementary material:**

The online version of this article (doi:10.1186/s12974-015-0396-3) contains supplementary material, which is available to authorized users.

## Background

Accumulating evidence suggests that neuroinflammation contributes to tauopathy, the pathological accumulation of tau proteins in neurodegenerative disorders such as Alzheimer’s disease (AD) [1]. The brain cells involved in AD-related neuroinflammation include microglia and astrocytes. Astrocytes, the largest and most abundant neuroglial cells in the brain, become activated in AD and contribute to a reinforced inflammatory cascade [2]. A variety of factors, including amyloid beta (Aβ) and proinflammatory cytokines, may activate astrocytes [2]. Reactive astrocytes in turn produce nitric oxide and proinflammatory cytokines such as interleukin-1 (IL-1) and IL-6, which accelerate tauopathy including formation of neurofibrillary tangles (NFTs) [3–5]. Although these findings suggest a possible link between neuroinflammation and tauopathy, there is little evidence for a direct role of reactive astrocytes in the pathological accumulation of the microtubule-associated protein tau.

Macrophage migration inhibitory factor (MIF) is a proinflammatory cytokine found in many tissues and in cells such as monocytes and macrophages [6]. MIF has multiple functions including catalytic activities [7], lymphocyte activation [8], glucocorticoid counter-modulation [9], and proinflammatory regulation [10]. In addition to modulating the immune system, MIF is involved in tumorigenesis [11]. However, the function of MIF in the brain is not well understood. MIF is found in neurons, astrocytes, and microglia of the hippocampus and cortex [12], and proinflammatory stimuli significantly upregulate the expression of neuronal MIF at both mRNA and protein levels [13]. Clinical studies have shown a marked increase in MIF concentration in the cerebral spinal fluid (CSF) of AD patients [14]. Another piece of evidence indicating MIF involvement in AD is the elevated expression of CD74, which is a receptor for MIF [12, 15]. MIF has also been functionally linked to the toxicity of Aβ aggregates, which is a major constituent of AD plaques [16, 17]. Despite these findings, a correlation between MIF and tauopathy has not been established experimentally.

Tau hyperphosphorylation is a major pathological feature of neurodegenerative disorders such as AD [18]. In this study, we evaluated the effects of MIF deficiency in tau hyperphosphorylation using two mouse models of AD, one involving intracerebroventricular (ICV) injection of streptozotocin (2-deoxy-2-(3-methyl-3-nitrosoureido)-d-glucopyranose; STZ) into WT and *Mif*^−/−^ mice, and the other being the APP/PS1 transgenic mice mated with *Mif*^−/−^ mice. The results have shown that MIF deficiency significantly attenuated tau hyperphosphorylation in these mouse models. Moreover, the expression of MIF in astrocytes and the number of reactive astrocytes were markedly increased in the AD models, whereas MIF deficiency is associated with decreased astrocyte activation in these mice. Finally, we found that conditioned medium from activated astrocytes could stimulate tau hyperphosphorylation in neurons in a MIF-dependent manner. Based on these findings, we postulate that MIF plays a role in tauopathy through activation of astrocytes.

## Methods

### Reagents

Primary antibodies used in this study are listed in Table 1. Dulbecco’s modified Eagle’s medium (DMEM), neurobasal-A and B-27® supplements were purchased from Gibco (Invitrogen, Carlsbad, CA). The BCA protein assay kit and 4,6-diamidino-2-phenylindole (DAPI) were obtained from Beyotime Institute of Biotechnology (Nantong, Jiangsu, China). Mouse and rabbit control IgGs were purchased from Santa Cruz Biotechnology (Dallas, TX). IRDye® 800CW secondary antibodies were from LI-COR (Lincoln, NE). Other chemicals were obtained from Sigma Chemical Company (St. Louis, MO).

**Table 1:** Primary antibodies used in this study

Antibody	Type	Specificity	Phosphorylation sites	Reference/source
Tau5	Mono-	Tau (total)		Invitrogen, Carlsbad, CA, USA
Tau1	Mono-	Tau (non-phos.)		Millipore, Temecula, CA, USA
pS199	Poly-	P-tau	Ser199	Invitrogen
pT205	Poly-	P-tau	Thr205	Invitrogen
pS396	Poly-	P-tau	Ser396	Invitrogen
pS404	Poly-	P-tau	Ser404	Invitrogen
GFAP	Poly-	GFAP		Proteintech, Chicago, IL, USA
GFAP−Cy3™	Mono-	GFAP		Sigma-Aldrich, St Louis, MO, USA
MIF	Poly-	MIF		Abcam, Cambridge, MA, USA
Anti-β-actin	Mono-	β-actin		Sigma
Anti-GAPDH	Poly-	GAPDH		GoodHere Technology, Hangzhou, China

### Mice and treatments

The *Mif*^−/−^ (MIF-KO) mice in C57BL/6 background were prepared as reported previously [19]. All mice were housed (four to five animals per cage) with a 12/12 h light/dark cycle, with ad libitum access to food and water. The housing, breeding, and animal experiments were in accordance with the National Institutes of Health Guide for the Care and Use of Laboratory Animals, with procedures approved by the Biological Research Ethics Committee of Shanghai Jiao Tong University. Breeding with the APP/PS1 mice were detailed in Additional file 1.

**Fig. 1:**
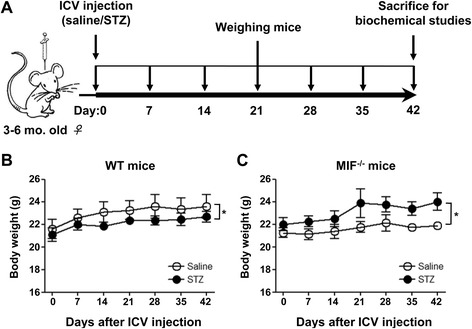
Schematic representation of study design and body weight change in *Mif* ^−/−^ mice and WT littermates. **a** Female mice aged 3-6 months received intracerebroventricular (ICV) injection of either saline or 3.0 mg/kg STZ on day 0. Mice were weighed once every 7 days until day 42, when mice were sacrificed and hippocampus, cerebral cortex and brain sections were prepared for further biochemical studies. The body weight of WT and *Mif* ^−/−^ mice receiving STZ or saline was recorded and shown in (**b**) and (**c**), respectively. Each group contained 6-8 mice and the data shown are the means ± SEM. *p < 0.05 compared with controls

Female WT and *Mif*^−/−^ mice of 3–6 months in age were randomly divided into control and ICV-STZ (streptozotocin, 2-deoxy-2-(3-methyl-3-nitrosoureido)-d-glucopyranose; Sigma) groups. The ICV-STZ mice were established by intracerebroventricular injection of a single dose of STZ [20]. Briefly, mice were anesthetized by intraperitoneal injection of 5 mg/ml pentobarbital sodium (Sigma) and then restrained onto a stereotaxic apparatus. Each mouse received a single ICV injection of 3 mg STZ/kg body weight, which was freshly prepared in normal saline, into both lateral brain ventricles. The bregma coordinates used for injection were −1.0 mm lateral, −0.3 mm posterior, and −2.5 mm below. As controls, ICV-saline mice received an equal volume of normal saline. All mice were placed on heating pads (37 °C) until recovered from surgery. The body weight of these mice was measured once every 7 days. Six weeks after ICV injection, all mice were sacrificed by decapitation and their brains removed immediately (Fig. 1a). The hippocampi and cerebral cortices of the mice were dissected, flash frozen in dry ice, and stored at −80 °C for biochemical analyses later. Some brain tissues were fixed with 4 % paraformaldehyde in 0.1 M PBS, followed by cryoprotection in 30 % sucrose. Sagittal sections of 30-μm thickness were cut using a freezing sliding microtome. The sections were stored in glycol anti-freeze solution (ethylene glycol, glycerol, and 0.1 M PBS in 3:3:4 ratio) at −20 °C until immunohistochemical staining.

### Western blot

Mouse brain tissue was homogenized in lysis buffer containing 50 mM Tris-HCl (pH 7.4), 100 mM NaF, 2 mM EDTA, 10 mM β-mercaptoethanol, 2 mM sodium vanadate, 8.5 % sucrose, 5 μg/ml aprotinin, 100 μg/ml leupeptin, and 5 μg/ml pepstatin. Protein concentrations were determined by using a BCA Kit according to the manufacturer’s protocol. Tissue homogenates were separated on 10 % SDS-PAGE, and separated samples were transferred onto nitrocellulose membranes (Whatman Protran®). The blots were incubated with primary antibodies and further treated with corresponding secondary antibodies before scanning using an Odyssey P140-CLx Infrared Imager (LI-COR Biosciences, Lincoln, Nebraska). Densitometric quantification of protein bands in Western blots was analyzed using the TINA software (Raytest Isotopenmessgeraete GmbH, Straubenhardt, Germany).

### Immunofluorescence staining

Sections of mouse brain were permeabilized in 3 % H_2_O_2_ and 50 % methanol in 0.05 M TBS (containing 0.05 M Tris buffer and 9 g/l NaCl) for 30 min at room temperature, followed by blocked with blocking solution containing 5 % normal goat serum and 0.1 % Tween-20 in Tris-buffered saline, pH 7.4 (TBS) at room temperature for 30 min. Sections were then incubated with primary antibodies overnight at 4 °C, rinsed with TBS, and further treated with secondary antibodies for 1 h in the dark at room temperature. Again after three washes in TBS, sections were stained for nuclei with 5 μg/ml DAPI for 10 min at room temperature, and then mounted on glass slides. Fluorescent images were taken on a laser-scanning confocal fluorescent microscope (Leica TCS SP8, Leica Microsystems, Wetzlar, Germany). The immunofluorescence intensity was quantified using ImagePro Plus Software (Media Cybernetics, Silver Spring, MD). The results were expressed as mean ± SEM based on a minimum of five individual fields. Student’s *t* test was used for statistical analysis with the Prism software (Ver. 5, GraphPad, San Diego, CA).

### Primary neuronal cultures

Neuronal cultures were established in cortices obtained from 1-day-old *Mif*^−/−^ or WT mice as described before [21] with minor modification. In brief, cerebral cortices were removed from the brains of mouse pups, the meanings and microvessels were removed, and tissues were minced with a sterile razor blade. Tissues were digested with 0.025 % trypsin (Sigma) and 0.01 % DNase I (Sigma) at 37 °C for 10 min. The cell suspension was filtered through a 200 mesh sieve, and cells were plated on poly-d-lysine (Sigma)-coated 24-well plates at a density of 5 × 10^5^ cells per well. Two hours later, the DMEM medium (containing 10 % FBS, 100 U/ml of penicillin, and 100 g/ml streptomycin sulfate) were replaced with neurobasal medium containing 2 % B-27® supplements for 2 days. Culture medium were changed to neurobasal with 10 % FBS and 3 μg/ml cytosine-β-d-arabinofuranoside (Ara-C, Sigma) in the following 2 days, and then again switched back to neurobasal medium containing 2 % B-27® supplements. Experiments were performed on days 7–8 after initiation of the culture.

### Primary astrocyte culture

Astrocyte cultures were prepared from 1-day-old *Mif*^−/−^ and WT mice as described before [22, 23]. Specifically, separated cells were cultured in poly-d-lysine-coated 75-cm^2^ flasks with DMEM medium (containing 10 % FBS, 100 U/ml penicillin, and 100 g/ml streptomycin sulfate). The medium was replenished on day 1 and day 3. On day 7, microglia cells in the culture flasks were shaken off at 260× rpm for 2.5 h, and the remaining astrocytes were maintained in DMEM with 10 % FBS until seeding into 6-well plates. Experiments were performed after one passage of the cells.

### Treatment with conditioned medium of astrocytes

When astrocytes cultured in plates grew to 70–80 % confluence, the culture medium was replaced with fresh DMEM without FBS, or with the above medium containing 75 mM or 150 mM glucose. The cells were incubated for another 12, 24, 48, and 72 h. At the end of the incubation, conditioned medium (CM) was collected and cells were lysed in 2×SDS buffer for immunoblotting analysis. The astrocyte CM collected at different time points was added to the culture of neurons from *Mif*^−/−^ or WT mice for another 12, 24, and 48 h. The neuronal cell lysate was prepared and analyzed by SDS-PAGE and Western blotting as detailed above.

### Statistical analyses

Data are presented as means ± SEM from at least three experiments. One-way ANOVA followed by Newman-Keuls test or Student’s *t* test was performed using the statistic software Prism 5 (GraphPad). *p* < 0.05 was considered statistically significant.

## Results

### MIF deficiency reverses body weight decrease in ICV-STZ mice

In this study, we used two mouse models of AD to investigate the effect of MIF on tau phosphorylation. The first model was generated by ICV injection of STZ (ICV-STZ) [24, 25] in *Mif*^−/−^ mice and WT littermates, using normal saline as a control (ICV-saline). As STZ-induced animal models are characterized by insulin deficiency, elevated glucose level, and lower body weight, changes in the body weight were monitored in these mice over a period of 42 days. Mice receiving ICV-STZ had lower body weight compared with mice receiving saline (Fig. 1b). This result was consistent with a previous report using rats [26]. In the *Mif*^−/−^ group, mice receiving ICV-STZ had higher body weight than those receiving ICV-saline (Fig. 1c). These results show that Mif deficiency reversed the decrease in body weight seen in ICV-STZ mice.

### MIF deficiency attenuates tau hyperphosphorylation in mice receiving ICV-STZ

**Fig. 2:**
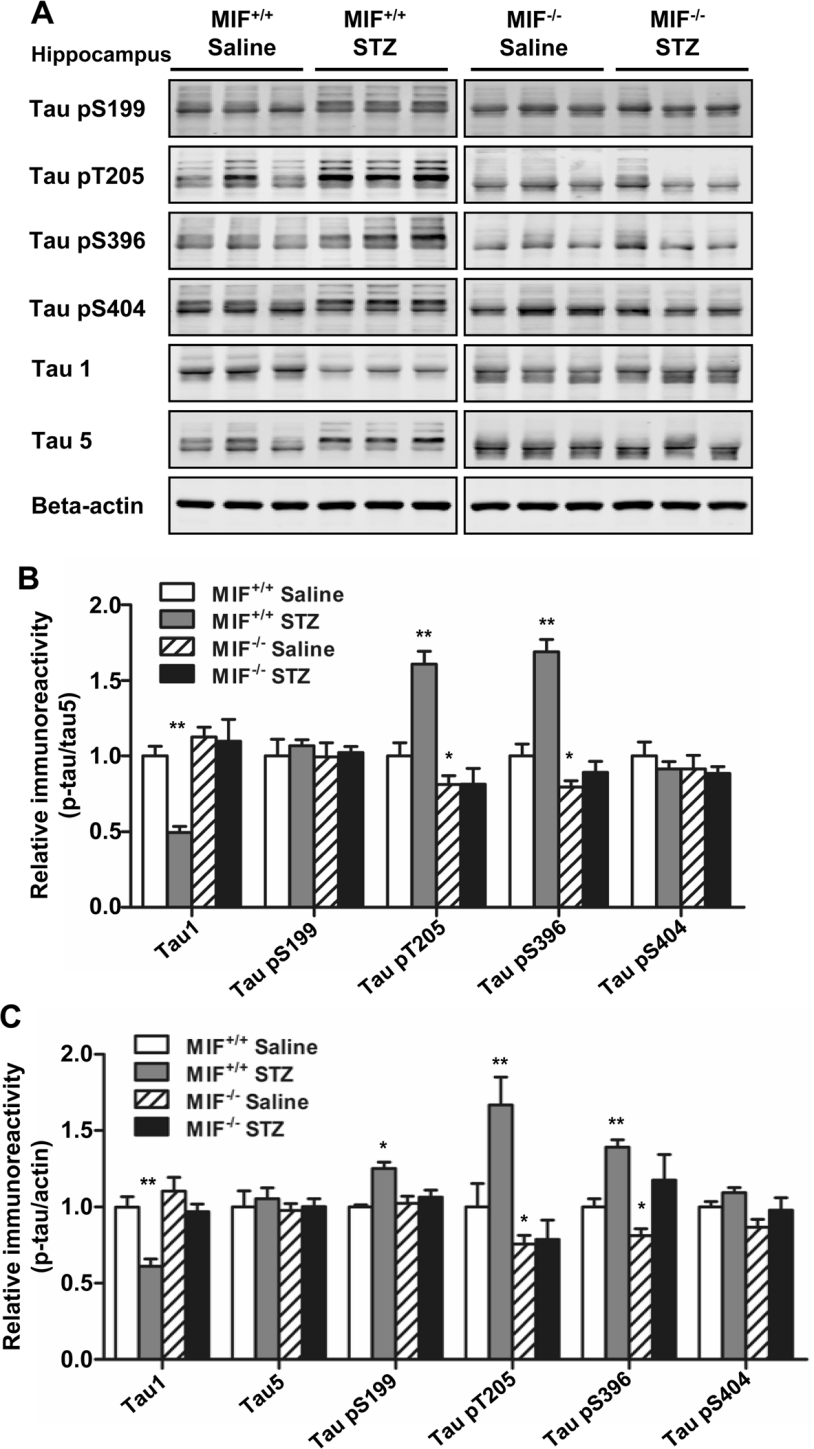
Effect of MIF on tau hyperphosphorylation in the hippocampus. **a** Representative Western blots showing tau hyperphosphorylation at S199, T205, S396, and S404 in the hippocampi of WT and *Mif* ^−/−^ mice receiving ICV injection of STZ or saline. The levels of non-phosphorylated tau (Tau1) and total tau (Tau5) were also measured. Quantification of the immunoreactivity of representative Western blots, normalized against total tau (**b**) and β-actin (**c**). *p < 0.05, **p < 0.01 compared with WT mice receiving saline

To investigate whether MIF affects tau phosphorylation, we examined the levels of total tau (using the Tau5 antibody), non-phosphorylated tau (using the Tau1 antibody), and phosphorylated tau at several AD-related amino acid positions including Ser199, Thr205, Ser396, and Ser404 in the hippocampus and cerebral cortex of mice receiving ICV injection of STZ. As expected, in the hippocampi of WT ICV-STZ mice, there was an increase in tau phosphorylation at Ser199, Thr205, and Ser396 (Fig. 2a, c) when compared with the ICV-saline controls. The same comparison also identified a marked decrease in the level of non-phosphorylated tau in WT ICV-STZ mice (Fig. 2a, c). When normalized against total tau protein (Tau5), an increase in tau phosohorylation at Thr205 and Ser396 was evident (Fig. 2b). In the *Mif*^−/−^ mice, however, no significant difference was observed in tau phosphorylation at these amino acids, nor was the expression of total tau and non-phosphorylated tau proteins different between the ICV-saline and ICV-STZ groups (Fig. 2a–c). Moreover, a comparison of the ICV-saline groups found that tau phosphorylation at Thr205 and Ser396 was reduced in the *Mif*^−/−^ mice (Fig. 2a–c). In cerebral cortex, increased tau phosphorylation was also observed at Thr205 and Ser396 in the WT mice receiving ICV-STZ, but tau phosphorylation at these sites was significantly reduced in *Mif*^−/−^ mice receiving ICV-saline or ICV-STZ (Additional file 1: Figure S1). These findings suggest that MIF is involved in basal (saline) and induced (STZ) phosphorylation of tau in the ICV mouse model.

**Fig. 3:**
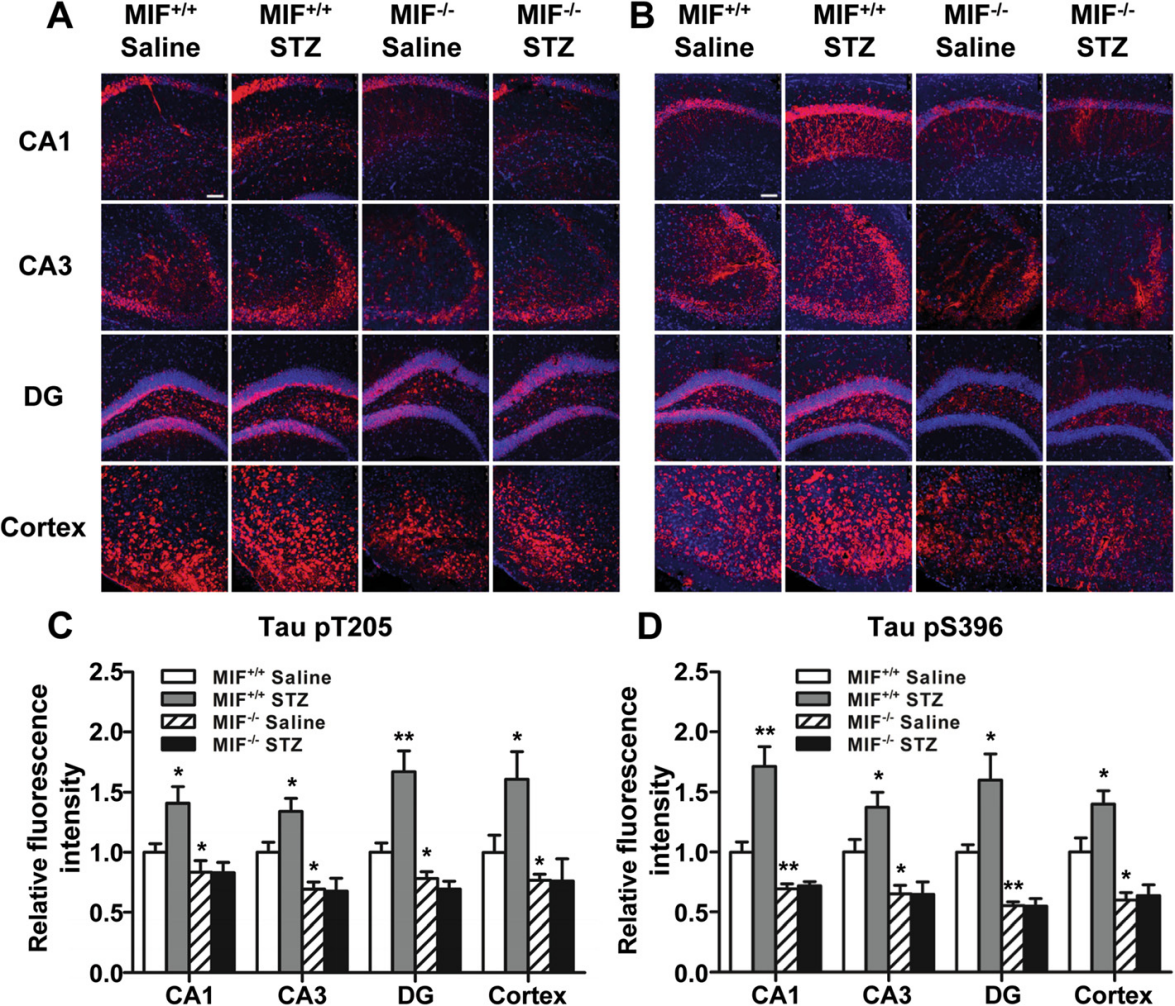
Staining of phosphorylated tau in brain slices from WT and MIF deficient mice. **a**, **b** Frozen slices from cortex and three different regions of hippocampus were stained for phosphorylated tau using rabbit polyclonal anti-Tau (pT205) and anti-Tau (pS396) antibodies. The sections were further treated with Alexa Fluor 568-conjugated anti-rabbit IgG (*red fluorescence*) and examined under a laser-scanning confocal microscope. Nuclei were stained with DAPI (*blue*). Scale bar in the upper left panes 75 μm (×200 magnification). Quantification of the level of tau phosphorylation at pT205 (**c**) and pS396 (**d**) in each of the regions, showing differences in fluorescence intensity between WT and *Mif* ^−/−^ mice receiving STZ and saline. Relative immunofluorescence was measured, and the results are presented as the means ± SEM based on five viewing fields for each region, using at least two mice. *p < 0.05, **p < 0.01 compared with *Mif* ^+/+^ mice receiving saline

In addition to immunoblotting, immunofluorescence staining was performed to confirm increased tau phosphorylation at Thr205 and Ser396 and their distribution in the brain. Frozen brain slices were stained with primary anti-Tau (pT205) and anti-Tau (pS396) antibodies, and then treated with Alexa Fluor® 568 conjugated anti-rabbit IgG (red). As shown in Fig. 3, increased red fluorescence was observed in the CA1, CA3, and dentate gyrus (DG) areas of hippocampus and in the cortex of ICV-STZ WT mice, compared with WT mice receiving ICV-saline. In contrast, no difference was observed between the STZ and saline groups in *Mif*^−/−^ mice. Similar to the results from immunoblotting analysis, absence of MIF lowered the baseline of phosphorylated tau at Thr205 and Ser396 in the brain slices. These results support the notion that MIF is involved in regulating tau phosphorylation in the mouse brain.

We also used the APP/PS1 double-transgenic mouse model to confirm MIF involvement in the regulation of tau hyperphosphorylation. APP/PS1 transgenic mice were mated with *Mif*^−/−^ mice. Six-month-old APP/PS1^+/−^*Mif*^+/+^ mice exhibited significantly increased tau phosphorylation at Ser199, Thr205, Ser396, and Ser404 when compared with age-matched APP/PS1^−/−^*Mif*^+/+^ control mice (Additional file 1: Figure S2). When mated with the *Mif*^−/−^ mice to generate APP/PS1^+/−^*Mif*^−/−^ offspring, tau phosphorylation at these sites was markedly attenuated in comparison with the APP/PS1^+/−^*Mif*^+/+^ mice (Additional file 1: Figure S2).

### MIF deficiency attenuates astrocyte activation in mice receiving ICV injection of STZ

**Fig. 4:**
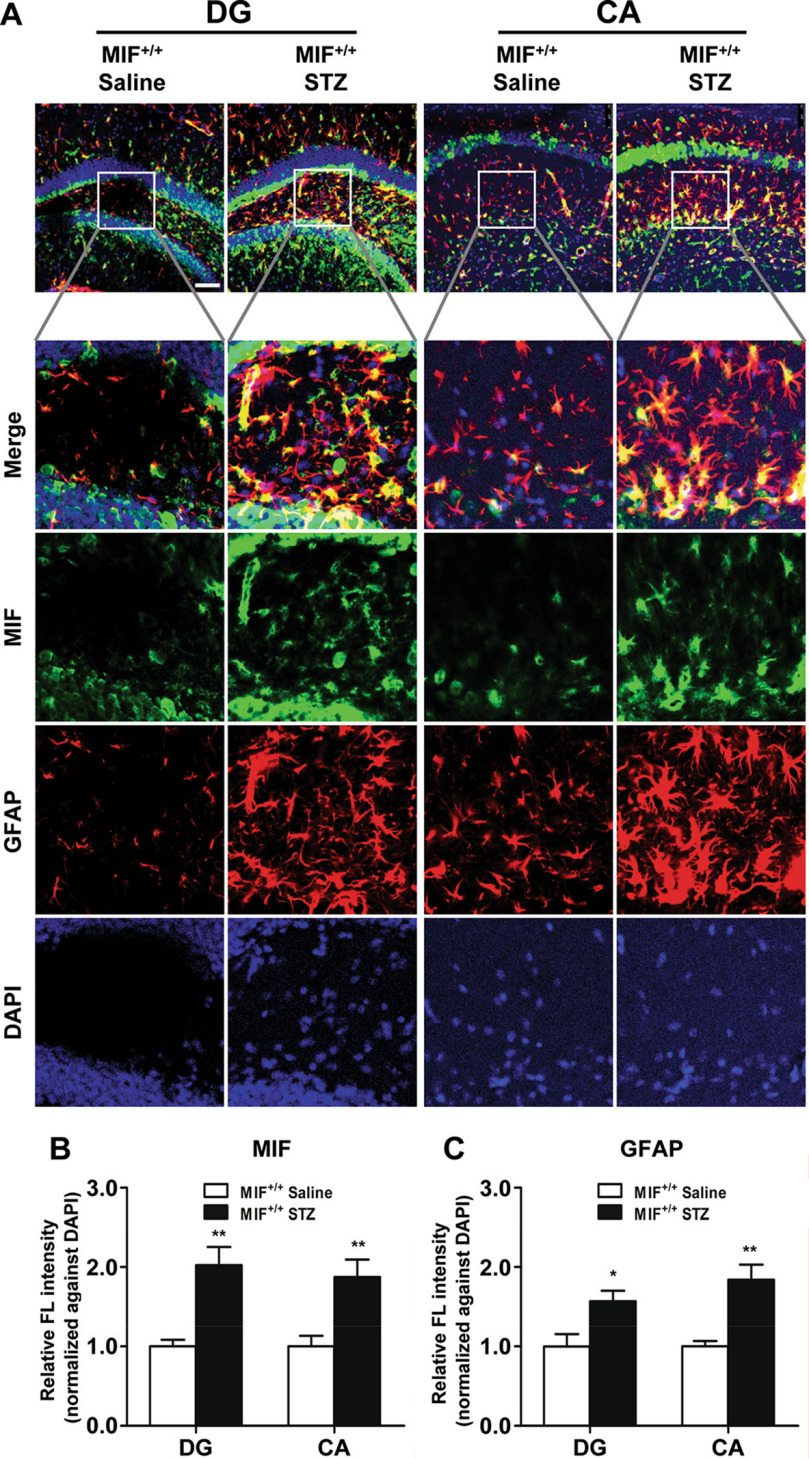
Upregulation of MIF in ICV-STZ mice and colocalization with GFAP in the hippocampus. **a** Serial sections of WT mouse brain were stained for MIF protein using a rabbit anti-MIF polyclonal antibody and Alexa Fluor 488-conjugated anti-rabbit IgG (*green fluorescence*). The sections were subsequently stained for GFAP using a monoclonal anti-GFAP Cy3^TM^ antibody (*red fluorescence*). Nuclei were stained with DAPI (*blue*). Images with combined fluorescent channels are shown at ×200 (scale bar in the upper left panel 75 μm). Selected areas are enlarged by 10 times and shown as combined as well as individual fluorescence stains. Quantification of the MIF (**b**) and GFAP (**c**) fluorescence was shown after normalization of the *green* (MIF) and *red* (GFAP) fluorescence against the *blue fluorescence* (nuclei). Data shown are means ± SEM based on multiple experiments (*n* = 3). *p < 0.05, **p < 0.01 compared with WT mice receiving saline

MIF is a proinflammatory cytokine that plays an important role in inflammatory response by stimulating glial cells. It is postulated that MIF-activated glial cells may contribute to tau phosphorylation in neurons. To test this possibility, we first examined the expression of MIF in WT mice that received ICV injection of saline or STZ. Immunofluorescence staining of serial slices from WT mouse brain with antibodies against MIF (green fluorescence) identified a significant upregulation of MIF in CA and DG areas of hippocampus in ICV-STZ mice compared to mice receiving saline (Fig. 4a, b). Moreover, it is evident that the increased MIF colocalized with neurons based on the staining pattern (Fig. 4a), consistent with a previous study [13]. To investigate the activation of astrocytes and their distribution, we stained frozen slices of brain tissue with CY3^TM^ conjugated mouse monoclonal anti-glial fibrillary acidic protein (GFAP; red fluorescence). The expression of GFAP, a protein abundant in activated astrocytes, was increased in ICV-STZ mice. Double immunofluorescence staining showed an increase in MIF colocalization with activated astrocytes in the brain of ICV-STZ mice (Fig. 4a), which showed increased GFAP expression (Fig. 4a, c). These results suggest that ICV injection of STZ induce the expression of MIF and the activation of astrocytes.

To further determine whether MIF is involve in the activation of astroyctes in the AD model, we compared WT mice with *Mif*^−/−^ mice that received ICV injection of saline or STZ for the expression of GFAP. Frozen brain slices from these mice were stained with the same anti-GFAP antibody. The results showed a marked increase in GFAP stain (red fluorescence) in the CA1, CA3, and DG areas of hippocampus and the cortex of WT mice that received ICV-STZ, compared to the ICV-saline group (Additional file 1: Figure S3A, S3B). In the *Mif*^−/−^ mice, however, there was no significant change in GFAP fluorescence between the ICV-STZ and ICV-saline groups. These results suggest that MIF is required for the activation of astrocytes in the ICV-STZ mouse model.

### MIF regulates tau phosphorylation through activation of astrocytes

**Fig. 5:**
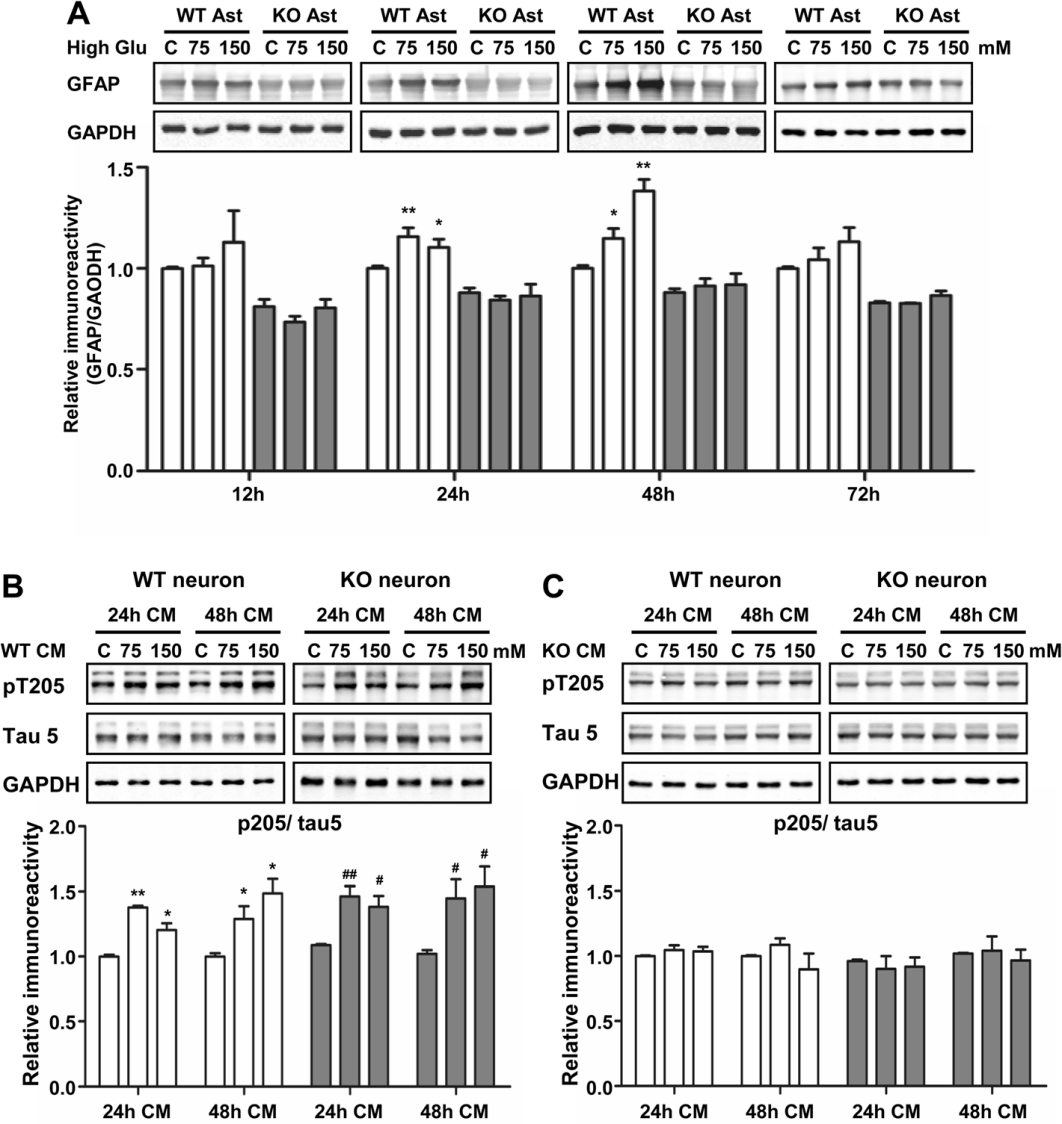
MIF regulation of tau phosphorylation through activated astrocytes. **a** Primary cultures of astrocytes (Ast) from WT and *Mif* ^−/−^ newborn mice were treated with DMEM with or without high glucose (75 mM and 150 mM) for 12, 24, 48, and 72 h. The cell lysate was analyzed by Western blotting for the expression of GFAP. The data are presented as densitometric values after normalization against the GAPDH level and are shown as the means ± SEM from at least three separate experiments; *p < 0.05, **p < 0.01 scompared with cells without stimulation. For **b** and **c**, freshly isolated neurons from WT and *Mif* ^−/−^ mice were cultured for 48 h with conditional medium (CM) of WT (**b**) and *Mif* ^−/−^ (**c**) astrocytes that were exposed to high glucose (75 mM and 150 mM) or normal medium (**c**, DMEM) for 24 and 48 h. Western blotting was performed to detect phosphorylated tau (pT205) in primary neurons. The blots were quantified by densitometry after normalization against total tau (Tau5). All data shown are means ± SEM of multiple experiments (*n* ≥ 3), each in dulplicate. **c** Control. * and ^#^ *p* < 0.05, ** and ^##^ *p* < 0.01 compared with medium without high glucose

To determine whether MIF acts on neurons directly, we examined the levels of tau phosphorylation in cultured neurons that were exposed to MIF and the MIF inhibitor ISO-1. None of these treatments had a significant effect on tau phosphorylation (Additional file 1: Figure S4A and S4B), suggesting that MIF might affect tau phosphorylation through an indirect mechanism. Since MIF is known to stimulate astrocyte activation, it was postulated that MIF regulation of tau phosphorylation might be mediated through activated astrocytes. Given that STZ injection led to astrocyte activation as well as an increase in glucose level [24], we sought to expose cultured primary astrocytes from WT and *Mif*^−/−^ newborn mice to high glucose (75 mM and 150 mM) to mimic the effect of STZ injection in mice. High glucose conditions have been used in previous studies at glucose concentrations up to 150 mM, but these high concentrations are suitable only for cultured cells [24, 27–29]. The level of GFAP in cultured astrocytes was determined by Western blotting and used as an indication of astrocyte activation. As shown in Fig. 5a, the expression level of GFAP in WT astrocytes was significantly increased after 24 and 48 h of high glucose treatment. However, this effect was absent in *Mif*^−/−^ astrocytes (Fig. 5a, shaded bars), suggesting that MIF plays a role in high glucose-induced activation of astrocytes.

High glucose treatment of neurons did not alter the level of tau phosphorylation significantly (Additional file 1: Figure S4C), nor did it significantly alter the viability of the cultured neurons and astrocytes (data not shown). To further examine the role of astrocytes in MIF-induced tau hyperphosphorylation, primary neurons from WT and *Mif*^−/−^ mice were cultured in the presence of CM collected from WT and *Mif*^−/−^ astrocytes exposed to high glucose. Under this experimental condition, primary neurons cultured with the CM from WT astrocytes showed elevated tau phosphorylation (Fig. 5b), whereas those cultured with CM from *Mif*^−/−^ astrocytes did not (Fig. 5c). Mif deficiency in neurons had no effect in the level of tau phosphorylation induced by the CM from high glucose-activated astrocytes (Fig. 5b, c).

**Fig. 6:**
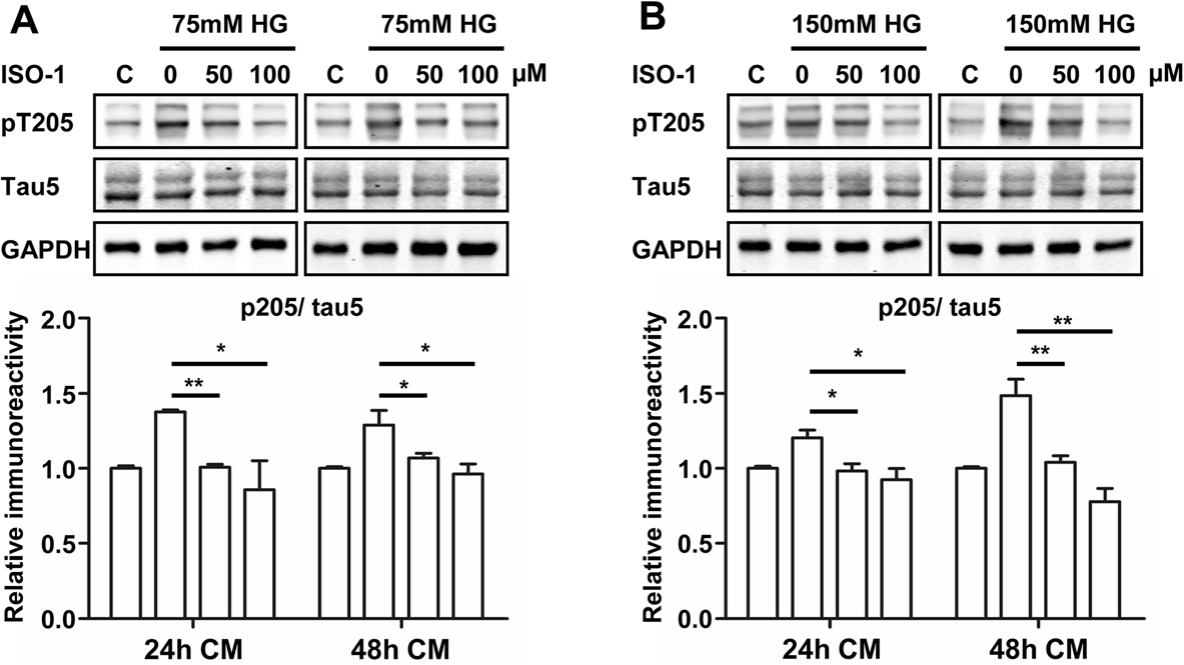
Inhibition of MIF during high glucose treatment of primary astrocytes abrogated the ability of the CM to stimulate tau phosphorylation in neurons. Primary astrocytes from WT mice were treated with 75 mM (**a**) or 150 mM (**b**) of high glucose in the presence or absence of the MIF inhibitor ISO-1 (50 μM and 100 μM). CM collected after 24 h and 48 h was added to primary cultures of mouse neurons. After 48 h, the neurons were harvested and the level of tau phosphorylation (pT205) was determined by Western blotting. Densitometry readings of the blots were normalization against those of total tau (Tau5). The level of tau phosphorylation in the untreated samples (DMEM without high glucose or ISO-1; marked as C in the first lane) was set at 1.0, with which other samples were compared. Data presented in bar charts are means ± SEM of three individual experiments. *p < 0.05, **p < 0.01 vs. high glucose medium without ISO-1

The role for MIF in high glucose-induced astrocyte activation was further tested using the MIF inhibitor ISO-1. When primary astrocytes were exposed to high glucose in the presence of ISO-1, CM collected from the cultured astrocytes could no longer stimulate tau phosphorylation at T205 in primary cultures of neurons (Fig. 6). Therefore, inhibition of MIF in astrocytes during treatment with high glucose abrogated the ability of the CM to stimulate tau phosphorylation. Collectively, these results suggest that MIF in astrocytes but not neurons plays an important role in elevated tau phosphorylation, which involves mediators released by the activated astrocytes.

## Discussion

The majority of published studies showed that intracellular NFTs often accompany activated astrocytes [30], suggesting possible links between activated astrocytes and tau phosphorylation. In one study, it was reported that astrocytes play an important role for Aβ-induced tau phosphorylation in primary neurons [31]. Another study showed that a small molecule, p75NTR ligand (LM11A-31), could attenuate tau hyperphosphorylation and activation of astrocytes in APP transgenic mice [32]. However, there has been no direct evidence for a role of astrocyte activation in tau phosphorylation. As a result, the identity of a suspected mediator remains obscure. The present study documents the effect of MIF in activating astrocytes and modulating tau phosphorylation, thereby providing novel insight into an indirect activation mechanism.

In this study, we evaluated the effects of MIF on tau hyperphosphorylation using two mouse models of AD. ICV injection of STZ is one of the well-known experimental models for sporadic AD [33]. We found that ICV-STZ injection into WT mice induced tau hyperphosphorylation at several AD-related sites including Ser199, Thr205, and Ser396 in the hippocampus and cerebral cortex. This result is consistent with two published studies that compared the ICV-STZ approach with other mouse models of AD [24, 34]. In *Mif*^−/−^ mice, no significant difference was found in the levels of tau phosphorylation between the ICV-STZ and ICV-saline groups. This finding provides first evidence for a potential role of MIF in STZ-induced tau phosphorylation. An involvement of MIF in maintaining the level of tau phosphorylation is also suggested by the observation that the baseline of phosphorylated tau at two of the AD-related sites (Thr205 and Ser396) was reduced in *Mif*^−/−^ mice.

Results obtained from another mouse model also support a potential role for MIF in regulating tau phosphorylation. The APP/PS1 transgenic mice have been widely used in studies of AD. Mutations in the genes of amyloid precursor protein (APP) and presenilins (PS1, PS2) increase the production of Aβ and cause familial Alzheimer’s disease. The APP/PS1 transgenic mice were mated with the *Mif*^−/−^ mice to generate APP/PS1/*Mif*^−/−^ mice for investigation of the potential role of MIF in tau phosphorylation. In these mice, an elevation in the level of phosphorylated tau, typically seen in the APP/PS1 mice, was attenuated in the absence of *Mif*. This result provides additional evidence for a role of MIF in tau hyperphosphorylation in the AD mouse. The mouse strain may be useful for further studies that will focus on the role of MIF on Aβ plaque formation and AD progression.

Different brain cells were analyzed for their involvement in MIF regulation of tau phosphorylation. No difference was found in the level of tau phosphorylation in primary cultures of neurons from either WT or *Mif*^−/−^ mice. Additionally, neither MIF nor the MIF inhibitor ISO-1 directly affected tau phosphorylation at T205 in primary neurons. Altogether, these results suggest that MIF indirectly regulates tau phosphorylation through other types of cells. Our finding that MIF increases GFAP expression suggests that MIF activates astrocytes. Based on this observation, we postulated that MIF regulates tau phosphorylation through activation of astrocytes. Our experimental results from transfer of astrocyte-conditioned medium to cultured neurons suggest that the MIF-activated astrocytes release mediator(s) that stimulate tau hyperphosphorylation in primary neurons. At present, the nature of the mediator(s) in the MIF-stimulated astrocyte medium remains unclear. Potential candidates include nitric oxide and S100B that are known to be released from astrocytes and induce tau hyperphosphorylation in cell culture-based experiments [5, 35]. Future studies will focus on these mediator(s) for their involvement in MIF-stimulated tau hyperphosphorylation through astrocytes.

## Conclusions

MIF is an inflammatory cytokine with pleiotropic functions in many chronic inflammatory diseases. Using two mouse models of AD, we demonstrate that MIF plays a role in the regulation of tau hyperphosphorylation in neurons. MIF does not directly affect tau phosphorylation; instead, MIF activates astrocytes that release mediator(s) capable of stimulating tau hyperphosphorylation in neurons. Our findings corroborate the notion that MIF may be a potential target for therapeutic intervention of AD.

## Additional file

Additional file 1: Figure S1. Involvement of MIF in tau hyperphosphorylation in the cerebral cortex of mouse brain. **Figure S2.** MIF regulation of tau hyperphosphorylation in APP/PS1 double-transgenic mouse model. **Figure S3.** Effect of MIF on astrocyte activation in mouse brain. **Figure S4.** Absence of direct effect of MIF, ISO-1, and high glucose on primary neurons. (DOCX 1588 kb)

## Abbreviations

AD: Alzheimer’s disease
APP: amyloid precursor protein
Aβ: amyloid beta
CM: conditioned medium
CSF: cerebral spinal fluid
DAPI: 4,6-Diamidino-2-phenylindole
DMEM: Dulbecco’s modified Eagle’s medium
GFAP: glial fibrillary acidic protein
ICV: intracerebroventricular
MIF: macrophage migration inhibitory factor
NFTs: neurofibrillary tangles
PS1: presenilin-1
STZ: streptozotocin
TBS: Tris-buffered saline
WT: wild type
