# Supplementary material for: Deficiency of macrophage migration inhibitory factor attenuates tau hyperphosphorylation in mouse models of Alzheimer’s disease
Source: J Neuroinflammation. 2015 Sep 17;12:177. doi: 10.1186/s12974-015-0396-3 (PMC4574615; doi:10.1186/s12974-015-0396-3)

### Supplementary Materials

**Supplementary Methods**

**Animals**

APP_SWE_/PS1dE9^+/-^ (APP/PS1^+/-^) mice in C57BL/6J genetic background were obtained from the Jackson Laboratory (stock # 005864). *Mif*^-/-^ mice were crossed to APP/PS1^+/-^ mice to generate APP/PS1^+/-^/MIF^+/-^ mice. The latter are further crossbred with MIF^+/-^ mice to create the following three genotypes: APP/PS1^-/-^/*Mif*^+/+^, APP/PS1^+/-^/*Mif*^+/+^, and APP/PS1^+/-^/*Mif*^-/-^. Mouse genotypes were determined by RT-PCR. Experiments were performed in accordance with the National Institutes of Health Guide for the Care and Use of Laboratory Animals, with procedures approved by the Biological Research Ethics Committee, Shanghai Jiao Tong University.

**Supplementary Figure Legends**

**Figure S1. Involvement of MIF in tau hyperphosphorylation in the cerebral cortex of mouse brain.** (A) Cerebral cortices dissected from the brain hemisphere of WT and *Mif*^-/-^ mice were homogenized in lysis buffer. Equal amount of homogenates were loaded to a 10 % SDS–PAGE for separation and samples were then blotted for multisite phosphorylated tau (pS199, pT205, pS396, pS404), non-phosphorylated tau (tau1) and total tau (tau5). (B, C) Relative immunoreactivity was obtained by densitometric quantification (mean ± SEM) of immunoblots, which was normalized against total tau (B) or β-actin (C). *, *p*<0.05, **, *p*<0.01, compared with WT mice receiving saline.

**
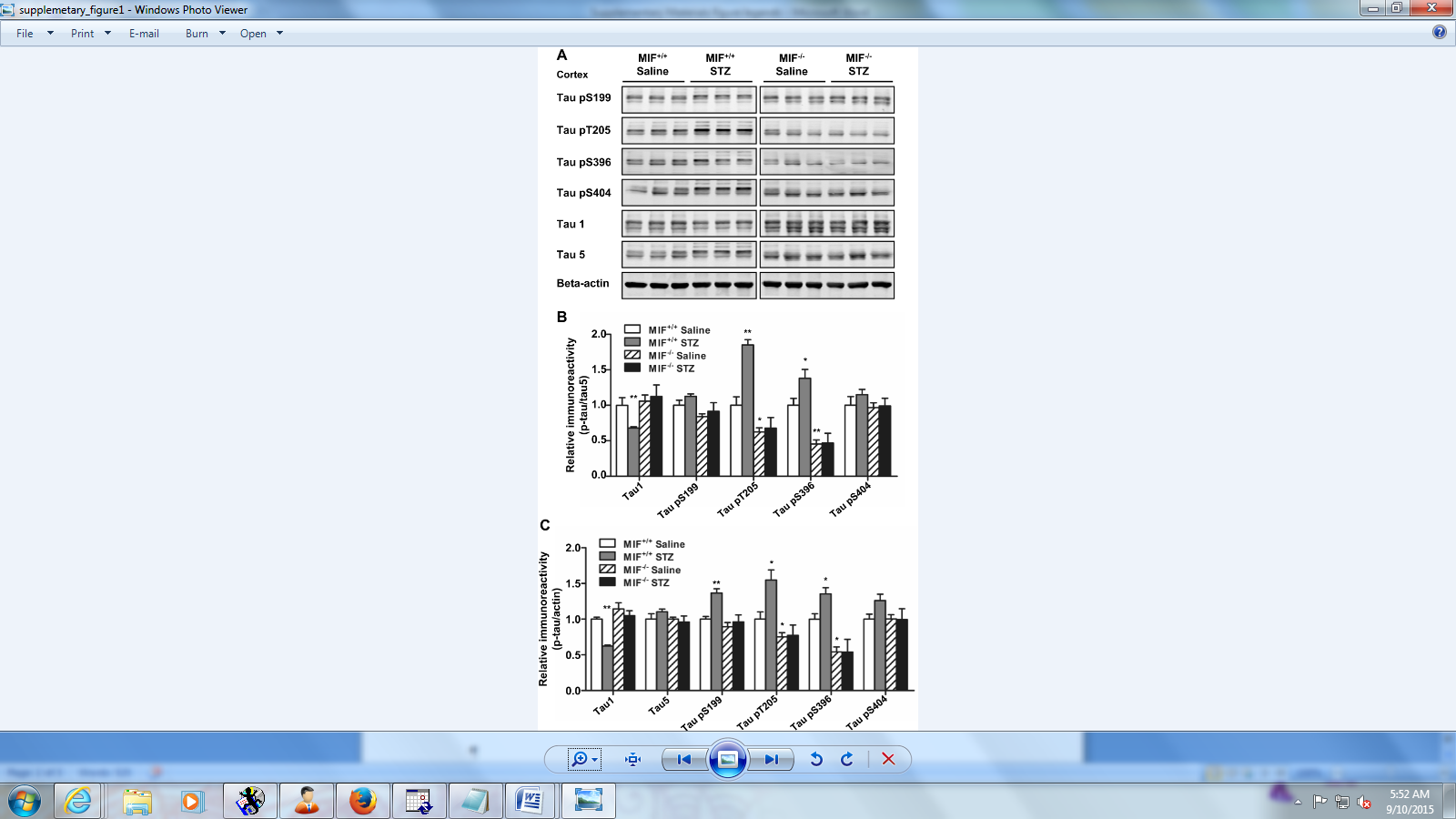
**

**Figure S2. MIF regulation of tau hyperphosphorylation in APP/PS1 double transgenic mouse model.** APP/PS1^+/-^ mice were mated with *Mif*^-/-^ mice to produce APP/PS1^+/-^*Mif*^-/-^ mice. Tau phosphorylation in hippocampus and cortex was evaluated by analyzing tissue homogenates using Western blotting. Anti-phosphor-tau antibodies (anti-Tau (pS199, pT205, pS396, pS404) and the monoclonal antibody Tau5 (for total tau) were used as indicated. Two mice were used in each group.


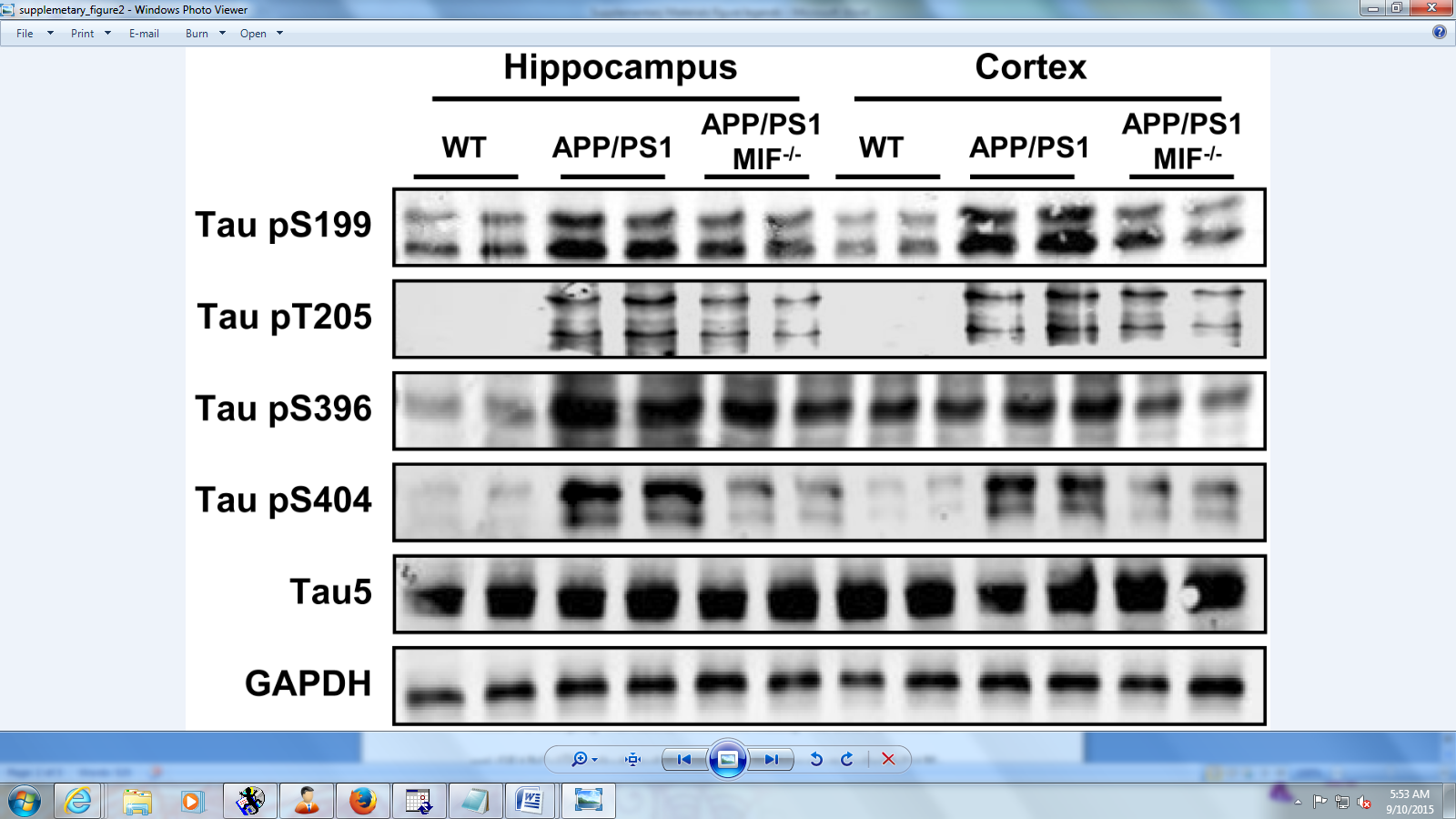


**Figure S3. Effect of MIF on astrocytes activation in mouse brain.** Activation of astrocytes was indicated by the expression of the marker protein GFAP. (A) Brain sections of each group were rinsed, blocked and staining with mouse monoclonal anti-GFAP Cy3^TM^ antibody (red) for astrocytes. Nuclei were stained with DAPI (blue). Images were taken on a confocal microscope. Scale bar in the upper left panel: 100 µm (200X magnification). (B) Quantification of GFAP level was conducted using the ImagePro Plus 6.0 software. The results are expressed as the means ± SEM from at least three mice per group, each in duplicates or triplicates. *, *p*<0.05, **, *p*<0.01 vs. saline-treated WT mice.

**
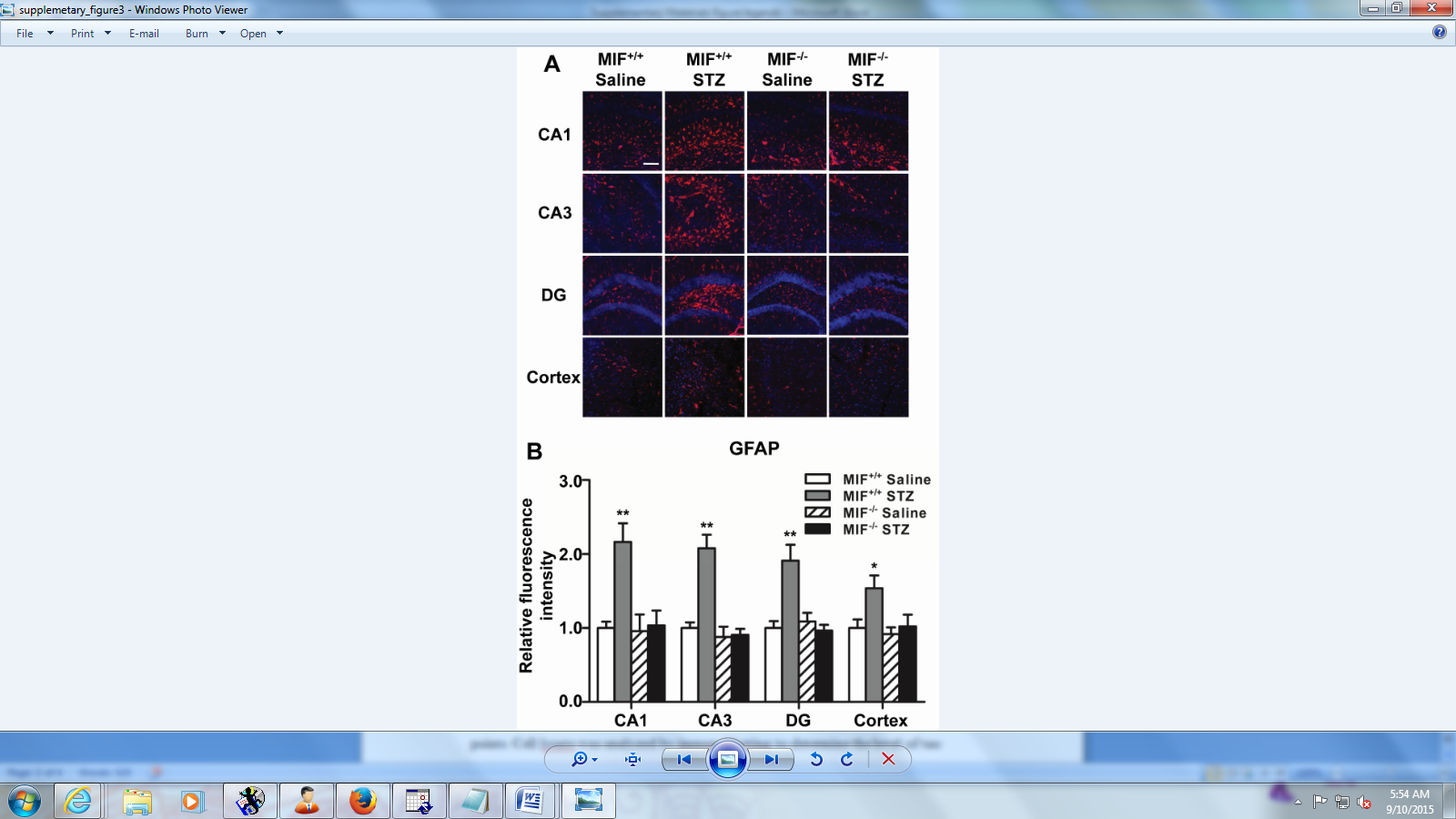
**

**Figure S4. Absence of direct effect of MIF, ISO-1 and high glucose on primary neurons.** (A) Primary cultures of neurons from newborn WT mice were exposed to different concentrations (2, 20 or 200ng/ml) of MIF at 37 ºC for the indicated time points. Cell lysate was analyzed by immunoblotting to determine the level of tau phosphorylation at site T205. The level of phosphorylation was quantified by densitometry and normalized against total tau (Tau5). (B) Cultured neurons from WT mouse pups were incubated with or without ISO-1 at indicated concentrations for 12 and 24h. The level of pT205-tau was determined as above in (A). (C) Cultured neurons from WT mouse pups were treated with DMEM with or without 75 and 150 mM glucose for 12, 24 and 48 h. C, control (DMEM only). Phosphorylation of tau at Thr205 was determined as above in (A). All data shown in this figure are means ± SEM from three separate determinations.


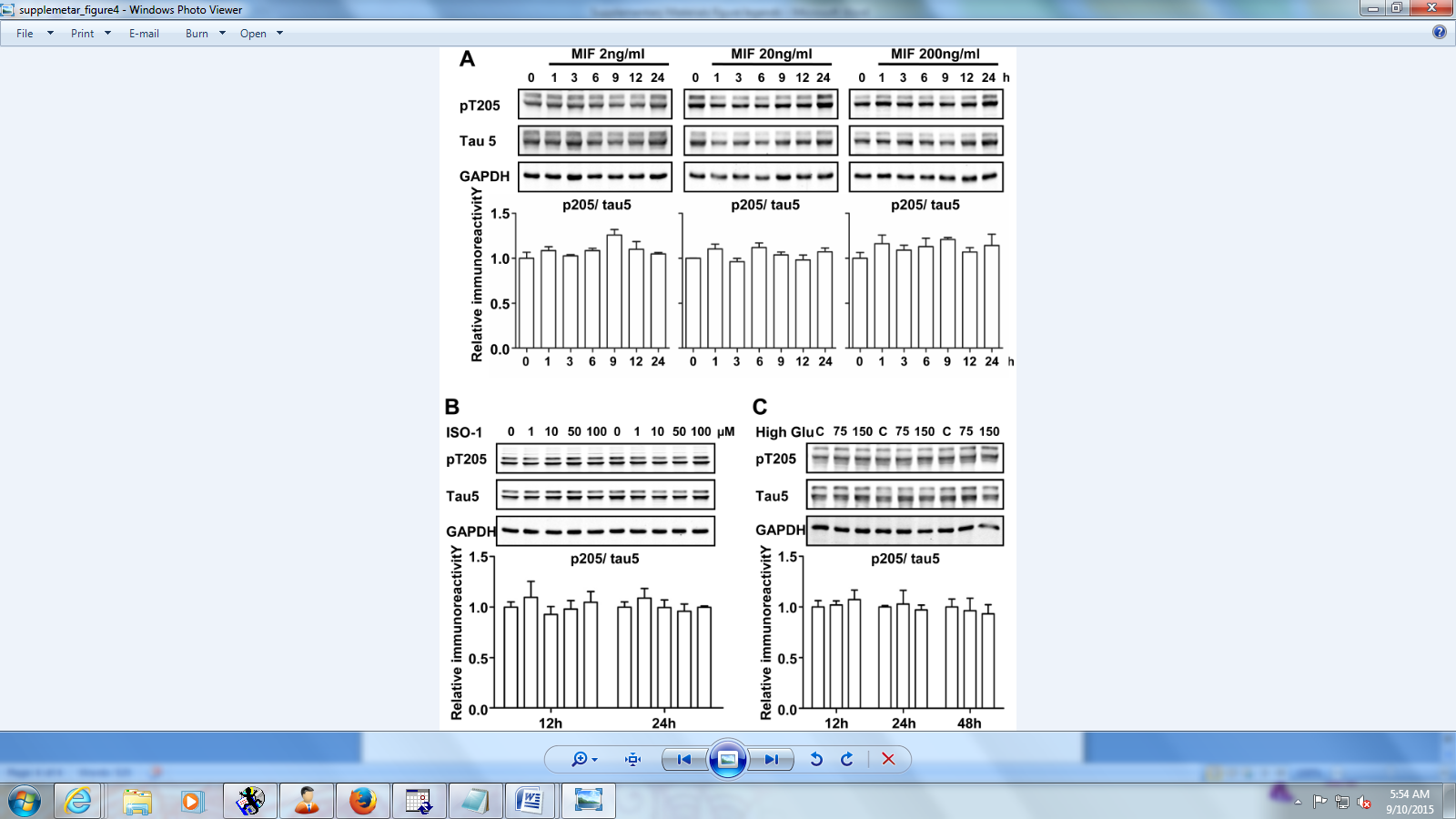

Supplement: Additional file 1: Figure S1. — Involvement of MIF in tau hyperphosphorylation in the cerebral cortex of mouse brain. Figure S2. MIF regulation of tau hyperphosphorylation in APP/PS1 double-transgenic mouse model. Figure S3. Effect of MIF on astrocyte activation in mouse brain. Figure S4. Absence of direct effect of MIF, ISO-1, and high glucose on primary neurons. (DOCX 1588 kb) [file 12974_2015_396_MOESM1_ESM.docx]
